# Routine cranial computed tomography before lumbar puncture in HIV-positive adults presenting with seizures at Mitchells Plain Hospital, Cape Town

**DOI:** 10.4102/sajhivmed.v16i1.354

**Published:** 2015-07-03

**Authors:** Salma Moolla, Ashmitha Rajkumar, Elma de Vries

**Affiliations:** 1 School of Public Health and Family Medicine, University of Cape Town, South Africa; 2 Western Cape Government Department of Health, Mitchells Plain Hospital, South Africa

## Abstract

**Background:**

Current international guidelines recommend that a cranial computed tomography (CT) be performed on all HIV-positive patients presenting with new onset seizures, before a lumbar puncture (LP) is performed. In the South African setting, however, this delay could be life threatening. The present study sought to measure the number of cranial CTs that contraindicate an LP and to predict which clinical signs and symptoms are likely to pose an increased risk from LP.

**Methods:**

The study was performed at a district level hospital in Western Cape Province. Data were collected retrospectively from October 2013 to October 2014. Associations between categorical variables were analysed using Pearson’s chi-squared test. Generalised linear regression was used to estimate prevalence ratios.

**Results:**

One hundred out of 132 patients were studied. Brain shift contraindicated an LP in 5% of patients. Patients with brain shift presented with decreased level of consciousness, focal signs, headache and neck stiffness. Twenty-five per cent of patients had a space-occupying lesion (SOL) (defined as a discrete lesion that has a measurable volume) or cerebral oedema. Multivariate analysis showed a CD4 count <50 (*p* = 0.033) to be a statistically significant predictor of patients with SOL and cerebral oedema. Univariate analysis showed focal signs (*p* = 0.0001), neck stiffness (*p* = 0.05), vomiting (*p* = 0.018) and a Glascow Coma Scale (GCS) < 15 (*p* = 0.002) to be predictors of SOL and cerebral oedema.

**Conclusion:**

HIV-positive patients with seizures have a high prevalence of SOL and cerebral oedema but the majority of them are safe for LP. Doctors can use clinical parameters to determine which patients can undergo immediate LP.

## Introduction

### Background

New onset seizures in HIV-positive adults have been reported to have an incidence of around 6%.^1^ Current guidelines, based on a number of international research articles,^2,3,4^ recommend that cranial computed tomography (CT) be performed on all HIV-positive patients presenting with new onset seizures, before a lumbar puncture (LP) is performed. Cranial CT, in addition to being a diagnostic tool, is used to advise if an LP can be performed safely. The concern about performing an LP in this group is the risk of brain herniation secondary to raised intracranial pressure.

Raised intracranial pressure is defined, in the acute setting, as pressure within the cranial vault that exceeds 20 mmHg – 25 mmHg for more than 5 minutes (Roytowski).^5^ Raised intracranial pressure *per se* has not been conclusively linked to the risk of brain herniation from LP.^3^ LP is in fact used to treat symptoms of raised intracranial pressure, particularly in patients with a communicating hydrocephalus. Common examples of cases where LP is therapeutic are idiopathic intracranial hypertension and cryptococcal meningitis (CCM).

Brain shift, however, has been associated with an increased risk of brain herniation from LP, and can be measured by a CT scan. Cranial CT can demonstrate hemispherical shift and gross generalised brain swelling, both contraindications to LP.^6^ Brain shift occurs when differences in pressure between brain compartments lead to areas of the brain being compressed against certain intracranial structures.^3^ The brain can literally be pushed to the point where it can herniate through the foramen magnum; this can result in death from compression on the medulla oblongata in the brain stem, which controls cardiac and respiratory functions.^5^ When performing an LP in patients with brain shift, downward pressure can increase and advance fatal brain herniation. Brain shift is usually caused by an expanding mass such as a space-occupying lesion (SOL), cerebral oedema or hydrocephalus.^5^

A prospective South African study performed at Chris Hani Baragwanath Hospital found that 53.3% of HIV-positive patients presenting with new-onset seizures had a SOL.^7^ Many clinicians are of the opinion that a suspected SOL is an absolute contraindication to LP before CT. Van Crevel et al.^3^ have shown, however, that herniation from LP can only occur when a SOL is accompanied by brain shift. Brain shift usually declares itself by clinical symptoms such as a decreased level of consciousness, headache and vomiting, neck stiffness, focal neurology, bradycardia and apnoea.^3^ The incidence of brain shift in an HIV-positive adult patient presenting with seizures is not known.

In developed countries, where CT scanners are readily available, it is feasible to scan all HIV-positive individuals presenting with seizures soon after presentation. In many South African hospitals, however, arranging a CT scan is time-consuming and is often not done after working hours. A working diagnosis and commencing treatment based on LP results can save time and lives whilst waiting for a cranial CT.

The purpose of the present study was to assess if current international guidelines that recommend a cranial CT before LP on all HIV-positive patients presenting with seizures, is applicable to the HIV-positive population of Western Cape Province, South Africa.

## Methods

This was a cross-sectional, observational study conducted at Mitchells Plain Hospital (MPH) from October 2013 to October 2014. MPH is a large metro district hospital providing level one (district level) as well as some level two (general specialist) care to approximately 440 000 people from the areas of Mitchells Plain, Phillippi and Crossroads. The study population comprised HIV-positive patients with seizures, presenting for cranial CT. The CT request forms filed in the Radiology Department at MPH were used to identify patients to be included in the study. Initially, a broad manual search for any patient whose cranial CT was requested for seizures, was undertaken. The search was then streamlined to only include HIV-positive patients and seizures. Where there was doubt regarding HIV status, confirmation was sought via the National Health Laboratory Service (NHLS) database. Patients under the age of 18 and patients whose seizures were acutely trauma related, were excluded from the study.

**FIGURE 1:**
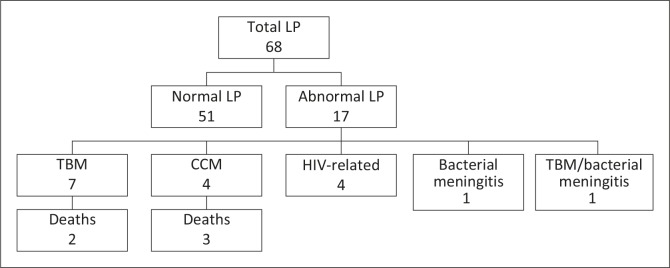
Lumbar puncture findings.

Data were extracted onto a data collection sheet from patient folders, the NHLS database and the Radiology Department database where necessary. Patients’ demographic details, CD4 count, risk factors for seizures, seizure history, seizure type, symptoms and signs of brain shift on presentation, CT information, LP findings (Figure 1), final diagnoses and prognoses were recorded. Criteria used for CT findings that would contraindicate an LP included any midline shift of the midline structures, effacement of any of the basal cisterns, and obliteration of the fourth ventricle. Diagnoses were the most likely diagnosis for the patient, recorded in the notes; this was not necessarily based on organism detection but also on doctors’ clinical impressions.

The data were then captured onto a Microsoft Excel spreadsheet and were analysed using STATA 13.0. Associations between categorical variables were analysed using Pearson's chi-squared test and prevalence ratios (PRs) with 95% confidence intervals (CIs). Variables that were considered as potential risk factors, such as age, gender, CD4, seizure history, type of fit and signs and symptoms of brain shift, were included in the model for ‘generalised linear regression analysis’ to estimate the PR. For all analyses, a *p*-value <0.05 and a 95% CI that did not span unity were considered the thresholds of statistical significance.

### Ethical considerations

Ethical approval was obtained from the University of Cape Town research ethics committee, and the Western Cape Provincial Health Research Committee granted permission to conduct the study. Patients’ identities were protected as their names were not recorded.

## Results

A total of 132 CT request forms, for the period October 2013 – October 2014, identified suitable patients to be included in the study. This figure suggests a minimum of 11 cranial CTs performed monthly at MPH on HIV-positive patients presenting with seizures. The first 100 patients whose folders were accessed were studied.

Baseline characteristics of patients are presented in Table 1. Patients’ ages ranged from 21–73 years, with the median age being 38. Twenty per cent (*n* = 20) of patients had advanced immunosuppression with a CD4 count <50. Patients who presented with seizures usually had some form of systemic illness such as TB or gastro-enteritis with renal impairment, which also placed them at risk for metabolic abnormalities, and hence seizures. New-onset, generalised seizures were most common. There were 42 patients who were asymptomatic at the time of presentation. Headache and a decreased level of consciousness were the most frequent clinical presentations. The presence or absence of papilloedema was recorded in 1 of the 100 folders. The assumption is that papilloedema was not checked for in the remaining 99 patients.

**TABLE 1:** Baseline characteristics of patients.

Characteristics	*n* and years
Age	
Median	38 years
IQR	32–45 years
CD4 count	
<50	20
50–200	23
200–350	24
>350	32
Unknown	1
Risk factors for seizures	
Systemic illness	38
Alcohol/substance use	21
History of head injury	13
Abnormal chemistry	8
Seizure history	
New onset seizure	81
Known epileptic	19
Type of seizure	
Generalised	74
Focal	16
Undocumented	10
Clinical signs and symptoms suggestive of brain shift	
Headache	21
Vomiting	4
Visual disturbances	1
GCS < 15	38
Focal signs	11
Neck stiffness	10
Papilloedema	None documented

*n*, portion of total sample (*N* = 100).

IQR, interquartile range; GCS, Glascow Coma Scale.

Brain shift was seen on CT scan in 5% of the patients. Details of these patients are given in Table 2. All of them had one or more signs and symptoms of raised intracranial pressure/brain shift on presentation. One patient with suspected meningitis had an LP before the cranial CT, which showed brain shift. No adverse effects were reported from the LP.

**TABLE 2:** Description of patients with brain shift.

Patient	CD4 count	Gender	Type of seizure	Symptoms	CT finding	LP done	Adverse effect from LP	Diagnosis	Prognosis
1	<50	Male	Generalised	Focal signs Impaired consciousness GCS 14	Active space-occupying lesion	No	Not applicable	Toxoplasmosis	Referred tertiary institution
2	50–200	Female	Focal	Focal signs Headache GCS 15	Active space-occupying lesion	No	Not applicable	Toxoplasmosis	Recovery and discharge
3	50–200	Female	Unrecorded	Impaired consciousness GCS 13	Generalised cerebral oedema	Yes, after second scan	No	Meningitis bacterial/TB	Recovery and discharge
4	200–350	Male	Generalised	Impaired consciousness Neck stiffness GCS not documented	Active space-occupying lesion	Yes, before scan	No	Tuberculoma/TB meningitis	Recovery and discharge
5	200–350	Female	Generalised	Impaired consciousness GCS 14	Localised cerebral oedema	No	Not applicable	Chronic haematoma/empyaema	Referred tertiary institution

**FIGURE 2:**
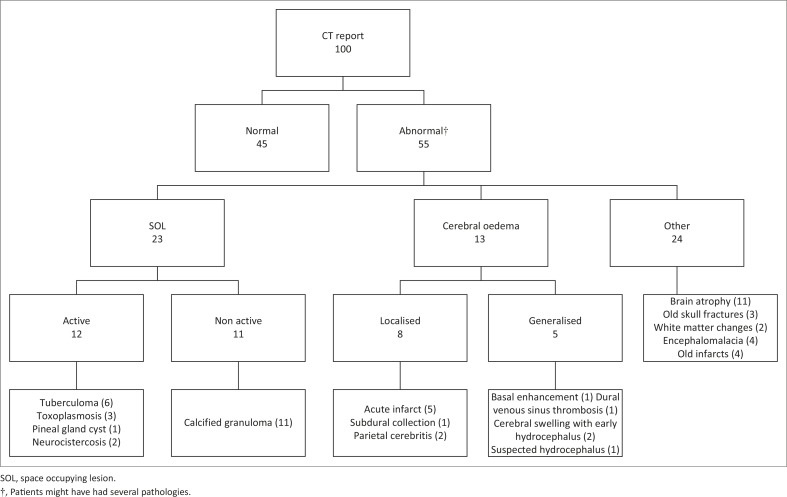
Computed tomography scan findings.

Results of cranial CTs are set out in Figure 2. More than half of the patients (*n* = 55) had an abnormal cranial CT. Many of these abnormalities were old and untreatable. Patients with active SOLs, together with the patients with cerebral oedema (generalised and localised), were at risk of developing brain shift. This proportion comprised 25% of the study population. Calcified granulomata were categorised as inactive SOLs as they have no potential for growth and expansion. In our setting, a calcified granuloma is attributed to old TB, but there are a number of different causes. Nearly a quarter (*n* = 24) of patients had brain abnormalities that might have explained their seizures but were incurable, such as old infarcts, calcified granulomata and focal encephalomalacia.

Generalised linear regression was used to predict patients with SOL/cerebral oedema (Table 3). Associations were initially found in the following categories: age, CD4 count, asymptomatic, focal signs, neck stiffness, vomiting and decreased level of consciousness. However, after multivariate analysis only, a CD4 count <50 was associated with an increased risk for SOL/cerebral oedema. All other variables such as age, gender, history of seizure, and type of seizure (whether localised or generalised) had no predictive value in at-risk patients. The group of patients with brain shift was too small to show any significant clinical predictors and they were therefore grouped together with patients with active SOLs and cerebral oedema.

**TABLE 3:** Univariate and multivariate analyses of potential predictors of patients with active space-occupying lesion and cerebral oedema.

Potential predictors for at-risk patients	Totalnumber of patients	Percentageof patients with active SOL or cerebral oedema	Univariate			Multivariate		
PR	95% CI	*p*-value	PR	95% CI	*p*-value
Age								
<40	58	32.8	2.3	1.0–5.2	0.0352	1.8	0.7–4.5	0.236
CD4								
> 350	32	3.1	1	-	-	1	-	-
200–350	24	25.0	2.3	1.4–3.8	0.0143	8.3	1.0–71.0	0.053
50–199	23	34.8	2.7	1.7–4.4	0.0017	7.5	0.9–62.0	0.063
<50	20	50.0	3.7	2.1–6.6	0.0001	10.1	1.2–85.4	0.033
Clinical presentation								
Asymptomatic	44	6.8	0.2	0.1–0.5	0.0002	0.3	0.1–1.3	0.118
Focal signs	11	72.7	3.8	2.2–6.7	0.0001	2.0	0.8–5.1	0.154
Neck stiffness	10	50.0	2.3	1.1–4.7	0.0543	1.0	0.4–2.8	0.995
Vomiting	4	75.0	3.3	1.7–6.4	0.0184	1.5	0.4–6.2	0.586
GCS < 15	38	42.1	2.9	1.4–5.9	0.0020	1.0	0.4–2.7	0.984

PR, prevalence ratio; CI, confidence interval; GCS, Glascow Coma Scale.

LPs were performed on 68% (*n* = 68) of patients presenting with seizures. Despite international guidelines, 52% of these patients (*n* = 35) had an LP before cranial CT, with no adverse effects reported. A markedly abnormal cerebrospinal fluid (CSF) result was found in 24% of patients (*n* = 16) who underwent LP, of whom 75% (*n* = 12) were diagnosed with infective meningitis. Meningitis comprised more than half of the total study mortality (5 out of 9 deaths). Other causes of death were from renal failure (2 out of 9 deaths) and gastroenteritis (2 out of 9 deaths).

The proportion of patients who did not have a clear diagnosis on discharge comprised 23% (*n* = 23), 13 of whom had an LP and 10 of whom did not have an LP. Sixteen per cent of patients had seizures attributed to the use of drugs or, alcohol, a previous head injury, a metabolic cause or a breakthrough seizure (in the known epileptic group).

## Discussion

Uptodate,^8^ the international evidence-based clinical decision support resource, has advised that patients with suspected meningitis be scanned first if they have any of the following: decreased level of consciousness, focal signs, papilloedema, preceding seizures and impaired cellular immunity. This recommendation was based on the study by Hasbun et al.^4^ By strict international standards, all patients in the present study ought to have had a CT scan before LP. Our study sought to measure the risk of LP in patients with preceding seizures and impaired cellular immunity by measuring the number of CT scans that reported brain shift and hence contraindicated an LP. It is the first study to look at the prevalence of brain shift and patients at risk for brain shift (SOL/cerebral oedema) in this particular subset of patients. An attempt was also made to predict if clinical factors in this group could be used to decide which patients needed a preceding cranial CT.

Despite the expected increased prevalence of an active SOL (12%) and cerebral oedema (13%) in the population studied, the actual number of patients who had a contraindication to LP on CT scan was small (5%). This finding prevented us from drawing any significant statistical conclusion from the descriptive results in Table 2. We were, however, able to show statistically significant associations, on univariate analysis, between clinical predictors and patients with an active SOL or cerebral oedema (Table 3). Active SOLs and cerebral oedema are disease processes that could lead to brain shift. It can therefore safely be assumed that patients with no clinical predictors of SOL and cerebral oedema will have a reduced probability of brain shift.

The present study confirmed recommendations that a decreased level of consciousness and focal signs are significant predictors of patients at risk for brain shift. It also found that vomiting and neck stiffness might also be positive predictors of SOL/cerebral oedema, in HIV-positive patients presenting with seizures. Of significance is the finding on multivariate analysis that a CD4 count <50 is associated with increased risk for SOL/cerebral oedema. A CD4 count <50 is a specific predictor and should be given more weight in clinical decision making. Two asymptomatic patients, with CD4 <50, underwent LP and subsequently had an active SOL on cranial CT. It must be stressed, however, that none of the asymptomatic patients was unsafe to LP.

Papilloedema has been described as a contraindication to LP. The patients in our study were almost entirely not examined for papilloedema; this may be owing to the fact that it is often difficult to perform ophthalmoscopy in a bright and busy emergency room, especially when doctors’ experience with ophthalmology is limited. Papilloedema, however, is a late finding of raised intracranial pressure, and guidelines from Queens University recommend performing LP even when the optic discs cannot be visualised,^9^ if there are no other contraindications to LP.

Extensive research was performed by the Swedish Infectious Disease Society regarding the comparative risk between immediate LP before CT and the risk of delayed LP (and inevitably delayed treatment) in adults with suspected acute bacterial meningitis. Hypothetical calculations of these risks, in different clinical settings with varying probabilities of cerebral mass lesions and acute bacterial meningitis (ABM), were presented. The authors worked on the premise that, although there is little evidence of an association between LP and brain herniation in acute bacterial meningitis, there is sufficient evidence of an association between LP and brain herniation in patients with cerebral mass lesions. The risk of brain herniation, associated with LP, in patients with cerebral mass lesions, however, is small and was assumed to be between 1% and 2%. It was concluded that where a patient had no clinical signs to indicate a SOL, an immediate LP will be advantageous; this would apply even where the probability of ABM is >0.5%. This research has led to the revised Swedish recommendation for early LP in 2009, which removed impaired immunity and new onset seizures as indications for a preceding cranial CT.^10^

An American study by O’Laughlin et al.^11^ examined 1737 CT reports in various patients with both medical and trauma-related presentations, to assess the prevalence of CT scans that contraindicated LPs. It was found that 14.6% had ≥ 1 high-risk findings that would contraindicate LP, compared with our study where 5% of CT scans contraindicated LP. Their study also found no clinical correlation between clinical presentation and CT findings. It did, however, draw the conclusion that because brain herniation precipitates death, radiologists are becoming increasingly cautious when reporting on CT scans. This assumption is made because actual brain herniation from LP is very rare, whilst the number of CT reports that contraindicate an LP is relatively common. To support this statement, there have been reports from casualty staff at MPH of patients undergoing LP before CT which later reported that LP was contraindicated. These patients, as with the one reported in the present study, had no adverse event after the preceding LP.

Cranial CT provided a diagnosis for seizures in more than 50% of our patients and it should remain an important diagnostic tool in our population. What is questionable is the need to provide our patients with urgent cranial CTs to rule out brain shifts before performing an LP. There are substantial financial and clinical implications that arise from this recommendation. The Hasbun study^4^ reported a 2-hour time delay to lumbar puncture and longer emergency department stays when patients underwent cranial CT before LP. In our setting, the time delays would be much longer, especially after hours when there is no CT service on site. The shortage of CT scanners and a lack of funding for staff has led to studies such as ‘The Kimberly hospital rule for urgent CT of the brain in a resource limited environment’^12^ and ‘Appropriateness of computed tomography and magnetic resonance imaging scans in the Eden and Central Karoo districts of the Western Cape Province, South Africa’.^13^ These studies, like ours, highlight the need to implement local, cost-effective CT guidelines.

Apart from the financial implications of arranging urgent CT scans, there is the clinical consideration. The highest number of deaths in our study was from CCM (3 out of 9 deaths), followed by tuberculous meningitis (TBM) (2 out of 9 deaths). The high case fatality rate in CCM, predicted by the WHO to be between 35% and 65% in sub-Saharan Africa prompted the recommendation that patients with a CD4 less than 100 have early screening for the disease.^14^ LP and CSF analysis remain the key diagnostic test for CCM but, if LP needs to be delayed, an urgent serum cryptococcal latex antigen test (CLAT) must be performed on all patients suspected of CCM.^15^ Patients with CCM often present with a severe headache and an isolated sixth nerve palsy. The *Southern African Journal of Infectious Diseases* (SAJEI) recommends that an LP be performed if meningitis is suspected despite the presence of isolated cranial nerve palsies.^6^ Current guidelines recommend blood culture analysis and IV antibiotics in cases of suspected meningitis where LP is contraindicated. The administration of IV antibiotics is intended to ensure that patients with bacterial meningitis are not deprived of emergency lifesaving treatment whilst waiting for lumbar puncture results. The prevalence of bacterial meningitis in our study was low in comparison with TBM and CCM. This is not unusual, as the causes of meningitis in a population with a high prevalence of TB and HIV, similar to our own, have already been described. The LP results of 4549 patients were studied between 2006 and 2008 at GF Jooste Hospital in the Western Cape and CCM followed by TBM were the most common causes of meningitis in this setting.^16^ Delayed LP will delay treatment in such patients and worsen their outcome.

### Limitations

The most significant limitation of the present study was the sampling strategy. Ideally, to measure the number of people who suffered immediate death after LP, it would have been necessary to identify all patients who had an LP. By identifying patients from their CT request forms, the study overlooked a possible group of patients who might have suffered immediate cerebral herniation post LP and never survived to have had a CT. Consultation with the Head of the Casualty Department, however, revealed that no patient, to his knowledge, suffered cerebral herniation from LP at MPH. Furthermore, at least 50% of our patients had an LP before CT and reported no complications.

The study design was adequate to report on the number of CT scans where LP was contraindicated. The small study sample and the small percentage of patients with brain shift prevented us from predicting any statistically relevant factors for brain shift. Enough information was available to describe these patients and it is not unreasonable to conclude that any decreased level in consciousness or focal signs, excluding isolated cranial nerve palsies, should contraindicate LP before CT in patients with HIV and seizures. Doctors working in hospitals with no CT scanners may benefit the remaining patients by performing LP before CT.

Another limitation is that our research conclusions are based on doctors’ clinical impressions and not necessarily measurable information. Neck stiffness, for example, is a subjective clinical finding and should not be used in isolation to decide on management steps. Similarly, patient diagnoses were not based on hard facts, owing to the low detection of organisms on CSF microscopy and culture as well as the absence of histology on SOLs. In such cases, doctors used their clinical judgement as well as evidence of disease elsewhere to make a diagnosis and start treatment. CCM and toxoplasmosis were, however, definitive diagnoses, based on positive India ink staining or cryptococcal antigen testing and positive toxoplasmosis serology, respectively.

The present study was a retrospective folder review, with all CT scans reported by the same radiologist, and might have been more reliable if the scans had been seen by two radiologists, to reduce any interpretation bias.

## Conclusion

HIV-positive patients with seizures have a high prevalence of SOLs and cerebral oedema but the majority of them are safe for LP. Indicators such as a decreased level of consciousness, focal signs, vomiting, neck stiffness and a CD4 count <50 should alert doctors to the possibility of at-risk patients. All the asymptomatic patients were safe for LP but should still have undergone non-urgent cranial CT owing to the limited occurrence of SOL and cerebral oedema in this group (6.8%). CCM accounted for the highest mortality, and doctors need to be more vigilant in performing serum cryptococcal latex antigen tests (serum CLATs) if LP is delayed. It is imperative that results of these tests be followed up promptly so that patients with CCM can be identified and treated early. There were no adverse events reported after any LPs performed on the patients in the present study.
